# Cases of Erysipelatous Laryngitis, or Black-Tongue

**Published:** 1845-10

**Authors:** A. M. Keller

**Affiliations:** Courtland, Ala.


### WESTERN JOURNAL

OF

### MEDICINE AND SURGERY.

OCTOBER, 1845.

ART. I.—*Cases of Erysipelatous Laryngitis, or Black-Tongue.* By A. M. KELLER, M.D., of Courtland, Ala.

In the history of the following cases, it has been my aim to give a faithful account of the *Black-Tongue*, or Erysipelatous Laryngitis, as I have had an opportunity of witnessing it in this neighborhood. I have reported every fatal case that occurred in my practice, and the treatment pursued in each. This has been unsatisfactory to me, but if I have apprehended the true nature of the disease, I have the authority of Cheyne, Ryland, Bayle, and others, for saying that it is one which is as fatal as it is rare in its occurrence. I very much regret that the prejudices of the community have prevented me from adding the information which might have been derived in these cases from post-mortem examinations. Imperfect as they are without this conclusion, I still hope that they will be found to add something to the history of this very formidable affection.

Case I.—This disease made its appearance first among us in the person of Mrs. T., aged about 25 years. She was attacked on the 7th of February with a severe rigor, pain in the head, back, and through the hips; having been very irregular in her menstruation for the previous seven months, her physician readily conceived her symptoms to arise from the menstrual effort, and treated her accordingly. I was called in the absence of her attendant to treat the case.

February 10th. Pain in the back, headache, and sore throat, externally on one side very much swollen, and great difficulty in swallowing; general uneasiness through the chest; hissing inspiration; cough; sensation of immediate suffocation. Upon looking into the throat, the top of the larynx, uvula, and fauces were found intensely inflamed and swollen, with patches of lymph adhering; pulse 120, and very compressible. Emetic of ipecac., friction to the throat, with vinegar, capsicum, blue mass, and rhubarb. Pediluvia of hot salt and water.

11th. Pulse 120; great thirst; tongue heavily coated with a dirty greyish fur; bowels freely operated upon; antimonial mixture every two hours; dilute muriatic acid gargle.

12th. Remission in all the febrile symptoms. Saline laxative, and a blister to the throat

13th. Symptoms aggravated; mercurial cathartic; antimonial mixture.

14th. Tongue perfectly black; fauces and nostrils stuffed with offensive exudations of lymph; difficult respiration; aphonia. Emetic of ipecac., mixture of turpeth mineral and ipecac. freely rubbed on the surface of the tongue; strong solution of nitrate of silver to the fauces.

I had the satisfaction to see the symptoms of throat disease all abate the 10th day; but in its place the true character of the disease manifested itself in an extensive erysipelatous eruption on the face, with general soreness of the whole surface.

Sulph. morphia to procure sleep; purgative of cal. and ipecac.; evaporating lotions to the erysipelas.

Unfortunately my patient proved to be three months advanced in pregnancy, and after a few uterine contractions a dead fœtus, putrid and offensive, was expelled. The placenta was not all removed at once, but came away piece-meal for two days. Irritative fever was set up, and this lady died in six days after the happening of abortion; but for this the above case would have terminated favorably. Every symptom of laryngitis was gone—tongue clean, respiration free and natural, deglutition easy.

Case II.—Miss E. P., aged 18 years, attacked with a chill, fever, and sore throat on the 7th of April.

9th. Tongue dry and heavily coated; fauces inflamed; difficult deglutition; spasmodic cough. Emetic of ipecac; mustard poultice to the throat; purgative of cal., ipecac. and pulv. ant.; muriatic acid gargle.

10th. Throat so much relieved that the patient ventured from her room and remained several hours.

11th. Relapsed. Violent return of throat disease, with all its attendant symptoms; pulse 120. Emetic of ipecac.; antimonial mixture; liniment of hartshorn, spirits turpentine, capsicum and lard.

12th. Erysipelas on face and scalp producing delirium, nervous excitement, and death on the 14th.

Case. III.—Negro girl Scilla, aged 13 years, attacked May 3d with chill, fever, and all the attendant symptoms of sore throat. Emetic of ipecac.; purgative of cal., ipecac., and scam.; muriatic acid gargle.

5th. Blister to the throat; solution of nit. potash, gum arabic, and sacch. album, every two hours; solution of nitrate silver to the fauces.

7th. Fever subsiding; throat better. This patient recovered, without any eruption, in a few days.

Case. IV.—Betsy, aged 35, attacked May 13th with chill, fever and sore throat.

14th. Pulse 110, full and strong; fauces much inflamed; epiglottis swollen; one side of the neck swollen and very painful. Venesection to 12 ounces; blister to throat; emetic of ipecac.; gargle of sage tea and alum; cal. and ipecac. at night.

15th. Castor oil and antim. mixture through the day, and morphia at night.

16th. Throat partially relieved; violent pain through the head; cold cloths applied constantly to the head, and a blister to the nape of the neck.

17th, 18th, and 19th. Pain in the head unabated; continue cold applications, and castor oil daily as a purgative.

20th. Excessive purulent discharge from both ears and the nostrils, which continued for a week so copious and offensive as to require fumigations of burning tar constantly in the room. This patient became very much prostrated from the discharge, but gradually recovered her health in six weeks under a mild tonic treatment, mild laxatives, and attention to the discharge. Hearing remains much impaired.

Case V. Carmichael, a negro man, aged 30, attacked on the 31st May with chill, fever, and sore throat; pulse quick and feeble. Treated with emetics, mercurial purgatives and a blister to the throat.

June 4th. Throat better; erysipelas on the face, and in 24 hours extended over the whole scalp. Constant delirium, picking at the bed-clothes. Upon consultation, venesection to 12 ounces was practised, and evaporating lotions to the head; blood in a few moments separated into a small quantity of serum, about two ounces of red crassamentum, and the remainder a yellow jelly-like substance—lymph. Died on the 8th of June. This patient, I was informed, after his death, had labored under syphilis for the three months previous to his last illness.

Case VI.—Kesiah, aged 25, attacked 1st of June with the usual symptoms of throat disease. Treated with emetics, antim. diaphoretics, purgatives of cal. and ipecac., and blister to the throat.

3d. Throat much relieved. Erysipelas on the whole of the right leg, pain intolerable; poultices and tepid affusions to the limb; cal. and ipecac. every three hours, after the fourth dose, purgative of castor oil. Cuticle cracked to the extent of four inches on the leg; offensive semi-purulent discharge. Intellect clear; swallows without pain; no indication of thoracic or abdominal disease. Died on the 7th. This patient also labored under syphilis for sometime previous to her attack of sore throat.

Case VII. Charles, aged 16, attacked June 1st with the usual symptoms of the epidemic. Treated with an emetic, blister to the throat, cal. and ipecac. quantities sufficient to act freely on the bowels. In two days convalescent.

4th. Relapsed from imprudence in eating. Erysipelas over the face and scalp; eyes entirely closed from the swelling; constant stupor; pulse 120. Blister to the nape of the neck; purgatives of calomel and scammony; slippery elm poultices to the face. Under this treatment the erysipelas gradually left him with intense soreness over the whole surface. Under the use of mild laxatives, and quinine occasionally, he recovered in a month and went to work.

Case VIII.—Sally, aged 45, of broken down constitution, attacked 6th of June with sore throat, accompanied with constant vomiting. Blister to the throat; cal., opium and ipecac. every three hours; stimulating enema next morning; solution of nitrate of silver to the throat. On the fourth day, without any amendment of the throat disease, erysipelas appeared on the face and scalp. On the sixth day, tongue black; varied applications of cold and warm water, and poultices of flax-seed; blisters to the nape of the neck. Mercurial purgatives daily until the 13th, when she became totally unable to swallow anything. Turpeth mineral and ipecac. to the tongue with no benefit; uvula entirely destroyed by ulceration. Died on the 16th day of disease.

Case IX.—Scott, aged 27, attacked with the usual symptoms, and was treated successfully with emetics, venesection, mercurial purgatives, and a blister to the throat. Went to the harvest-field and labored for three days, was caught in a shower of rain. Erysipelas occurred on the same day over the face and scalp. Treated by cupping to the back of the neck, mercurial purgatives, and occasional opiates, cold affusions to the head, all of temporary benefit, however, and this patient died after lingering two weeks. No evidence of thoracic or abdominal disease. The latter may have been obscured by the brain affection.

Case X.—William, aged about 30, has labored under syphilis for several months, attacked July 8th with slight throat disease. Treated by an emetic and purgative; poultice of capsicum to the throat; walked about the yard for three days and supposed himself well. I was requested to see him on the night of the 14th; violent pain and erysipelatous swelling of the left thigh; no soreness of throat or other symptoms of the epidemic. Pulse 100; venesection to 10 ounces; calomel and opium; poultices constantly applied to the limb.

15th. Symptoms all aggravated; no mitigation of pain; patient begged to have his leg cut off, and let him die easy at least: cupping and blisters to the limb. Repetition of calomel and opium, with castor oil; no relief, however, and this patient died of excessive pain on the morning of the 16th, 36 hours after the relapse.

I have notes of 20 other cases on the same plantation on which the last seven cases above occurred, which recovered. Four had the erysipelatous eruption on the face alone: one had it on both his legs, and the remainder had sore throat without any eruption; some recovering in five or six days; and others, in which the bronchia and lungs were involved, recovering in from two to three weeks.

Case XI.—Mr. S., aged about 28, attacked on the 31st May with the usual symptoms of the epidemic. Treated with an emetic, purgative, mustard poultices, and astringent gargles for two days. On the 4th and 5th June he rode into town. Relapsed on the evening of the 5th. Pulse 90; slight sore throat.

7th. Erysipelas on the face, and extending over the scalp in 24 hours. This patient became a perfect fright from the swelling, and size of the head, and the offensive discharge from the whole surface of the face. Treated with occasional purgatives of blue mass and ipecac., cal. and opium, antim. diaphoretics, the warm bath, blister to the back of the neck, flaxseed poultices to the face, and varied applications of cold and warm affusions to the head. The pulse never rose higher than 100 during the whole period of his illness. For several days he was so delirious as to require restraint in order to keep him in his house. He finally recovered, and on the 1st July was able to walk across his room.

+ Case XII.—Mrs. S., is the last, and by far the most interesting case I have to notice. She has labored for several years under liver disease, and in her general appearance presents much of the scrofulous diathesis. She was attacked on the same day with her husband, with the same symptoms of the epidemic. Treated by venesection, emetic of ipecac., blue mass, and ipecac. as a purgative; mustard to the throat. In three days she was so much relieved as to be able to walk about the yard.

June 4th. Relapsed. Erysipelas on the face on the 5th. Gave birth, on the morning of the 6th, to an eight months' child. This patient vacillated between life and death for a week. She was treated in the usual way for the erysipelas, which declined on the face and made its appearance on the hips and loins. Its progress was partially arrested by blistering in advance, but it terminated in large abscesses—one in the gluteal region: one at each hip joint: one in each groin: one under the chin: one under the eye, and one on the right side of chest. From as near an estimate as could be made, the three first-named abscesses discharged every twenty-four hours a quart of unhealthy fœtid matter, for three weeks. The abscess on the gluteal region discharged for one month and a few days.

Every means that could be suggested, by four physicians, was used to arrest this destructive discharge. Internally, mild laxatives, infusion of sarsaparilla, tincture iodine, hydriod. potash, port wine, and a sustaining diet. The roller bandage to both legs. She became so helpless and feeble, that it was a matter of life and death with her to submit to the necessary means of comfort and cleanliness. The local treatment consisted of bark poultices, stimulating injections, nit. silver, tinct. aloes, and myrrh, &c. Death took place on the forty-ninth day after the attack. She was too weak to be moved at all for two days previous to her death, and lay for two weeks a mass of flesh—loathsome to herself and almost intolerable to her friends. Upon minute examination after her death, I found the whole gluteal region sloughed away on one side—the ilium and sacrum exposed. The abscesses which pointed at the hips had burrowed, downwards, nearly to the knees, and communicated with the one on the ilium above. The infant died in a month after its birth of erysipelas of the face.

All the cases I have reported, with the exception of the first two, occurred on the same farm. All who waited on the sick had the disease in one or the other form, and from its whole history in this family, it certainly assumes the character of an infectious disease. It was carried to this plantation in the person of a negro from a farm six miles distant. No local cause could be discovered to originate the disease; but after its appearance, its character became more malignant, from the excessive heat of the season, and the crowding together of negroes in small and ill ventilated cabins.

A large open shelter was erected about a quarter of a mile distant in a grove, and all who could be spared from the sick were removed to it. The old cabins were thorougly renovated, and washed with lime. In about ten days the negroes were moved back again, and only two more cases of the disease occurred.

In its incipient stage the disease appears to be manageable in most cases, by emetics and stimulating applications internally, and externally to the throat. A relapse almost invariably terminated in the erysipelatous eruption. In a few cases among children, the eruption was miliary and easily managed.

Courtland, Ala., August, 1845.

